# The multi-tiered medical education system and its influence on the health care market—China’s Flexner Report

**DOI:** 10.1186/s12960-019-0382-4

**Published:** 2019-07-05

**Authors:** Chee-Ruey Hsieh, Chengxiang Tang

**Affiliations:** 1 University of Nottingham Ningbo China, 199 Taikang East Road, Ningbo, 315100 China; 2 School of Public Administration, Guangzhou University, Guangzhou, 510320 China; 3 National School of Development, Peking University, Beijing, 100871 China

**Keywords:** Physician, Medical education, Barefoot doctor, China

## Abstract

**Background:**

Medical education is critical and the first step to foster the competence of a physician. Unlike developed countries, China has been adopting a system of multi-tiered medical education to training physicians, which is featured by the provision of an alternative lower level of medical practitioners, or known as a feldsher system since the 1950s. This study aimed to illustrate the impact of multi-tiered medical education on both the equity in the delivery of health care services and the efficiency of the health care market.

**Methods:**

Based on both theoretical reasoning and empirical analysis, this paper documented evidence upon those impacts of the medical education system.

**Results:**

First, the geographic distribution of physicians in China is not uniform across physicians with different educational training. Second, we also find the evidence that high-educated doctors are more likely to be hired by larger hospitals, which in turn add the fuel to foster the hospital-center health care system in China as patients choose large hospitals to chase good doctors. Third, through the channels of adverse selection and moral hazard, the heterogeneity in medical education also imposes costs to the health care market in China.

**Discussion:**

Overall, the three-tiered medical education system in China is a standard policy trade-off between quantity and quality in training health care professionals. On the one hand, China gains the benefit of increasing the supply of health care professionals at lower costs. On the other hand, China pays the price for keeping a multi-tiered medical education in terms of increasing inequality and efficiency loss in the health care sector. Finally, we discuss the potential policy options for China to mitigate the negative impact of keeping a multi-tiered medical education on the performance of health care market.

## Introduction

Physicians are the captains of the health care team, indicating that whether physicians are competent has an important implication on the quality of health care services. Medical education is the first step to foster the competence of a physician, although learning by doing (on-the-job training) and other channels such as continuing education are also important to assure that physicians remain competent. In most high-income countries, physicians are a homogeneous group in that they receive the same level of education training no matter where they practice. In China, however, physicians are heterogeneous in that physicians differ in their educational training. Some receive full training in their medical professional education program while others only receive a short or partial training; hereafter, we refer to this as the multi-tiered medical education system.

The purpose of this paper is to explore the potential impacts of the multi-tiered medical education system from the perspective of health economics. Specifically, we investigate the effects of the multi-tiered medical education system on the performance of health care market in terms of equity and efficiency. We address two research questions: first, to what extent that the heterogeneity of medical education influences the equity in the delivery of health care services in China, and second, what kind of costs, in terms of both equity and efficiency, that China needs to pay for maintaining such a multi-tiered medical education system.

This study contains the following sections. In the next section, we first provide an institutional background on China’s medical education system. The key institutional feature is that China has adopted a multi-tiered education system to train physicians after since the 1950s. The “Methods” section demonstrated the analysis strategy and empirical methods. The “Results” section revealed the long-term impacts of medical system on the performance of health care market. The “Discussion” section documented the pros and cons of the medical education system, provided explanations from economic perspectives, and discussed mitigation policies. The final part presents a summary of the major findings and contributions.

### Institutional background

#### Overview

In China, there are substantial differences in the quality of medical education due to the existence of a three-tier education program. The first tier is degree-oriented medical education or bachelor program which includes 5 years of undergraduate medical studies followed by 3 years of residence. The second tier is still a tertiary medical education, or called junior medical colleges which provide 3 years of study after high school and lead to a vocational diploma. The third tier is a secondary-level medical education, or named secondary vocational schools which provide very limited medical training after junior middle school and lead to a secondary vocational diploma (SVD) [1–4]. Some doctors may receive a master or doctoral degree by extending the year of their training after the undergraduate study. For simplicity, we combine these two groups of doctors into the group of bachelor degree and refer them as a group of “bachelor degree or above.”

The current law allows medical graduates with a non-bachelor degree to become assistant doctors, and then they have the chance to obtain the full doctor licensure after they accumulate certain years of work experience and pass an examination [3, 4]. Thus, at a given point in time, the pool of Chinese physicians contains three types of physicians with different levels of education training; hereafter, we refer to these variations in education training across doctors as heterogeneity of medical education.

We further differentiate two types of heterogeneity in physicians in China from perspective of health economics, because, in the milestone paper of health economics, Arrow reported that four variables are deemed most important in the market of physicians: price of physicians (or wage rate of physicians), quantity of physicians, quality of entering medical students, and quality of medical education [5]. It is for certain that at least two critical variables, both the quality of entering students and quality of medical programs, substantially varied. First, medical students admitted to the bachelor program and junior medical college program are significantly different in terms of their quality. Like many other countries, graduates from high school who attempt to receive tertiary level education need to take the national college entrance examination (NCEE); however, those scores for bachelor programs in medicine are much higher than that for junior medical colleges. For example, only students who basically rank top 40% in NCEE have admissions into medical bachelor programs for the whole country, so junior medical colleges have to recruit from students ranked below 40% in NCEE [6]. Second, students who attend secondary vocational schools out of junior middle school have only a minimum 9 years of basic education, which brings about the quality problem of the entering students on the one hand; those secondary vocational schools in medicine generally offer limited and tailored courses to meet demands of those students on the other hand.

**Fig. 1:**
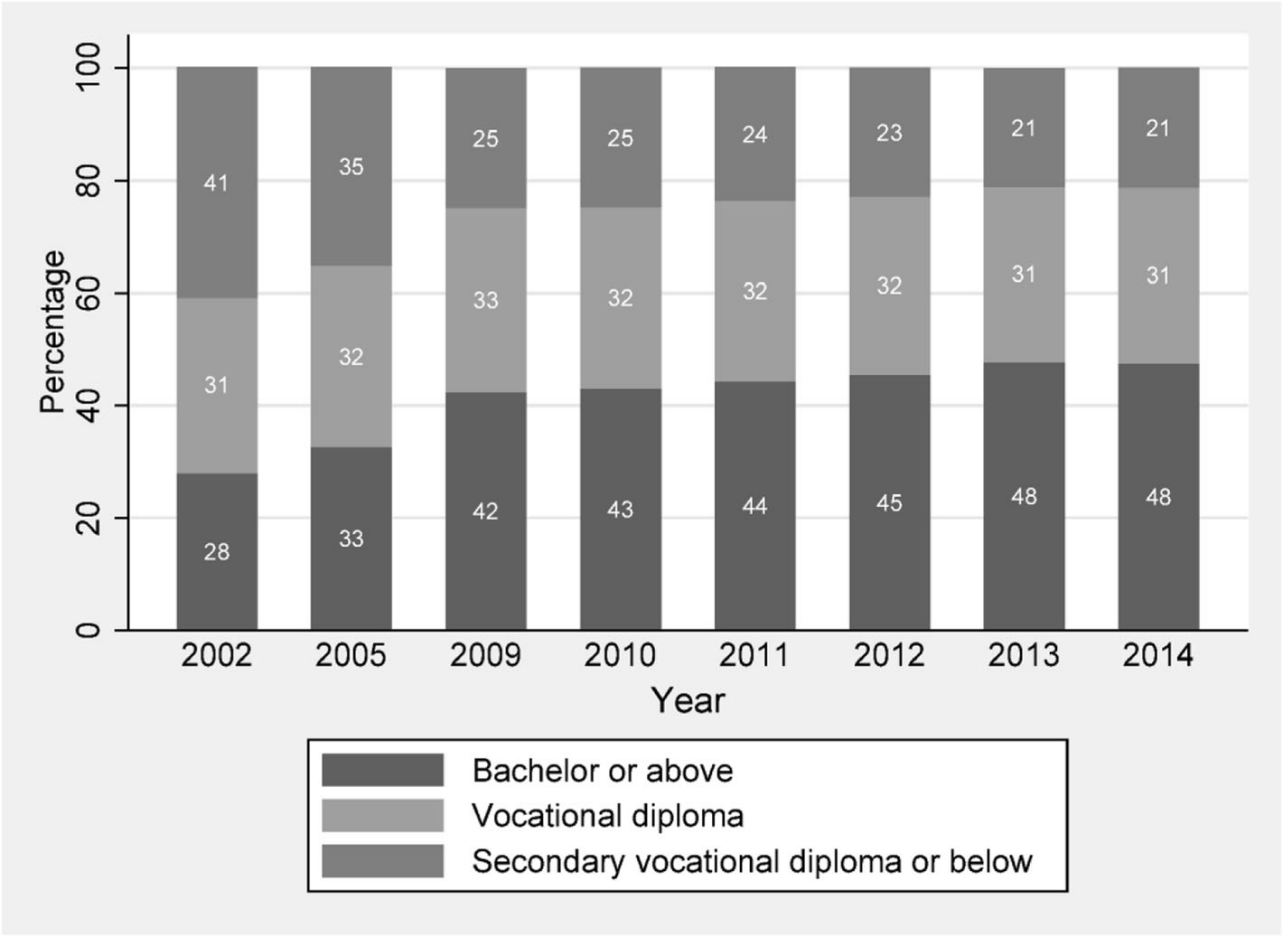
China’s doctors and assistant doctors by education level, 2002–2014. Sources: Health Statistical Yearbook of China (various years). Notes: (1) Data for 2003–2004 and 2006–2008 are not available. (2) Bachelor or above refers to the category that doctors have educational achievement high than a bachelor degree, including bachelor, master, and doctorate degrees. Vocational diploma refers to a junior college degree (Zhuan Ke) or a junior tertiary education, generally including 3-year post high school education; secondary vocational diploma or below refers to both secondary vocational diploma (Zhong Zhuan) and high school education

Figure 1 illustrates the distribution of physicians by education level between 2002 and 2014. Although the share of physicians with bachelor degree or above has increased over time, from 28% in 2002 to 48% in 2014, there are still more than half of physicians in China who do not receive the full entry-level medical training according to the current international standard. Entry-level medical education programs are tertiary-level courses undertaken at a medical school. Depending on jurisdiction and university, these may be either undergraduate-entry (most of Europe, Asia, South America, and Oceania) or graduate-entry programs (mainly Australia, North America); therefore, gaining a basic medical degree may take from 5 to 8 or even 9 years (please refer to: https://www.who.int/hrh/education/en/).

To be specific, in 2014, 48% of physicians in China possess a bachelor degree or above, 31% of physicians received medical training through junior medical colleges, and about one in every five physicians only received medical education through a much-abbreviated training program (SVD). The substantial variations in the years of medical education program across physicians in China suggest that the Chinese doctors are not a homogenous group. Rather, they are substantially heterogeneous in that one doctor is not the same as the other doctor in terms of quality of enrolled students and educational training.

#### Historical perspective on physicians’ supply

Given the fact that more than half of currently practicing physicians in China do not receive sufficient education training, an important question to be asked is whether this is a short-run or long-run phenomenon. If this is a short-run phenomenon, the heterogeneity of medical education is not a big concern as the current situation will be phased out very soon. By contrast, the heterogeneity of medical education in China is a serious public concern if this is a long-run phenomenon in that the physicians who do not receive the sufficient education training will persistently prevail in the market in a significant share. In order to address this question, we need to first look at the evolution of medical education in China (Table 1) and then to have more information on the annual number of enrolled medical students, medical graduates, and newly licensed physicians.

**Table 1:** The evolution of medical education in China

Time period	Events (major contents of education reform)
1949	Adopted the Soviet model of autonomous medical universities.
1966–1976	Culture revolution: medical education was disrupted.
1998	Introducing education reform that stand-alone medical institutions and schools were merged into comprehensive universities.
2009	The government adopted a reform plan that aims to phasing-out 3-year medical program at secondary level.
2013	The government initiated a plan to “standardize” the quality of medical education by launching a new system called 5+3: 5-year undergraduate study plus 3-year residency.

After the communist revolution, China followed the Soviet model of medical education, which highlighted both independent medical schools and a feldsher practitioners system. Feldsher is derived from the German Feldscher, which was coined in the fifteenth century. In the eighteenth century, the term was then exported with Prussian officers to Russia. There is clear evidence on the relationship between physicians and feldsher; however, no evidence shows that feldshers will gradually disappear while physicians with “formal” medical training are increasing in numbers [7]. In the vast majority of former Soviet countries, feldsher provides primary, obstetric, and surgical care services in rural and remote areas, which featured by a secondary-level medical education with a rather shorter period of training than the standard MD program.

**Fig. 2:**
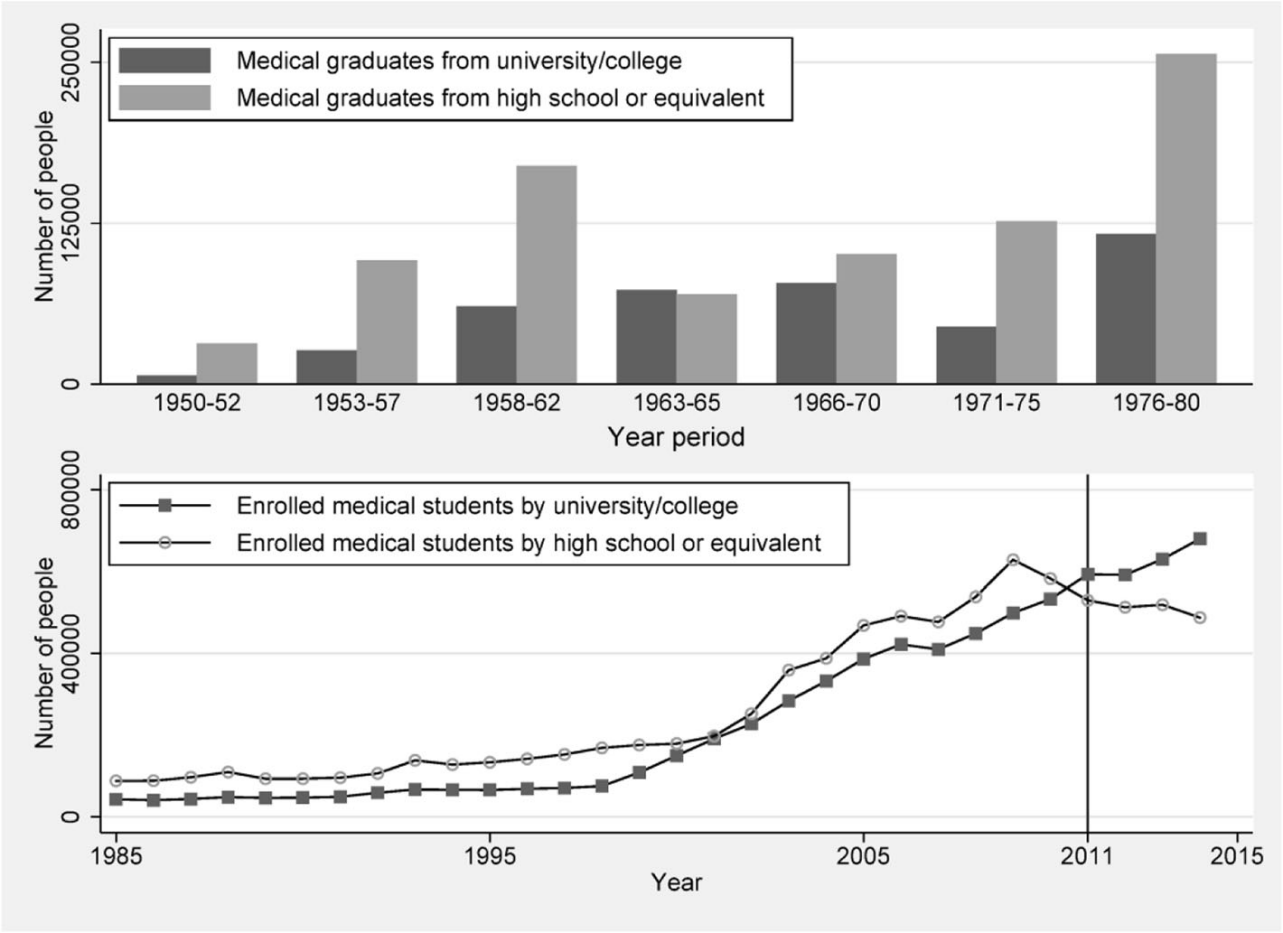
Time trend of medical graduates by education level, 1950–1980, and enrolled medical students by education level, 1985–2014. Source: Health Statistical Yearbook of China (various years)

The upper panel of Fig. 2 shows the overtime pattern in the number of medical graduates from secondary and tertiary medical schools during 1950 and 1980. Before 1980, the supply of medical students from tertiary school (university/college) had been only half of those students from secondary school for the most of period, with an exception between years 1963 and 1965. Before the market-oriented reform, China implemented a central planning economy, in which medical students obtain health care job by rationing entry. Therefore, the huge supply of middle-level medical students forms the majority of doctors before 1980. Even up to 2002, those doctors who only had secondary-level training in medical education still account for 41% of all doctors in China (see Fig. 1).

In the lower panel of Fig. 2, we depict the time trend of the number of enrolled medical students by education level from 1985 to 2014. It seems that China’s government realized that the previous model of medical education system cannot be sustainable; the government attempted to shut off the secondary education for medical professionals since 2011. From 2011 and so on, the number of enrolled medical students by tertiary institutions began to surpass those from secondary education. Along with the transition from a rationing entry system to a market with licensed practicing, it is becoming extremely difficult for medical students with only secondary-level training to be doctors, even though some secondary medical programs (secondary diploma, e.g. in rural medicine, TCM, and medicine of ethnic minorities) still exist and can be allowed to get practice license after examination. A particular policy needs to be pointed out is that a 5-year medical program after junior middle school was started and boosted from 2004 across western China, but was abolished in 2014 [8, 9].

#### Health care and medical education reform

During the last two decades, the government in China proposed two waves of major health care reform and followed corresponding medical education reform (Table 1). The first wave of reform started in 1998, which aimed to merge those independent medical institutions and colleges into comprehensive universities and thus expanded medical graduates rapidly to address profession shortage [10]. Due to the national health reform initialized in 2009, the ministry of education and ministry of health launched a comprehensive reform on medical education to improve both the distribution and enhance the quality of health workforce in 2012 [11]. Different from the 1998 reform, the principal objective of the 2012 reform is to improve the quality of doctors instead of increasing the quantity of human resources for health. The assignments of the second reform since 2012 include implementing physician education and training program of excellence, the establishment of accreditation system of medical program among universities, and the merge of professional master degree (master of medicine, MM) program and standardized residency training (SRT). The excellence plan is to build a common schedule for clinical doctors, called 5+3, in which 5 years of undergraduate medical education combined with 3 years of SRT will become a minimum requirement for a future practicing physician in China. The major progress of the latest reform is that the government aimed at eliminating 3-year medical programs at the secondary education level, which has been illustrated by the lower panel of Fig. 2. However, the disadvantage of the reform is that the government still discriminately supplies heterogeneous medical professions, e.g., medical graduates from 3-year tertiary vocational diploma, which are deemed as the mainstream source of rural health workforce.

**Fig. 3:**
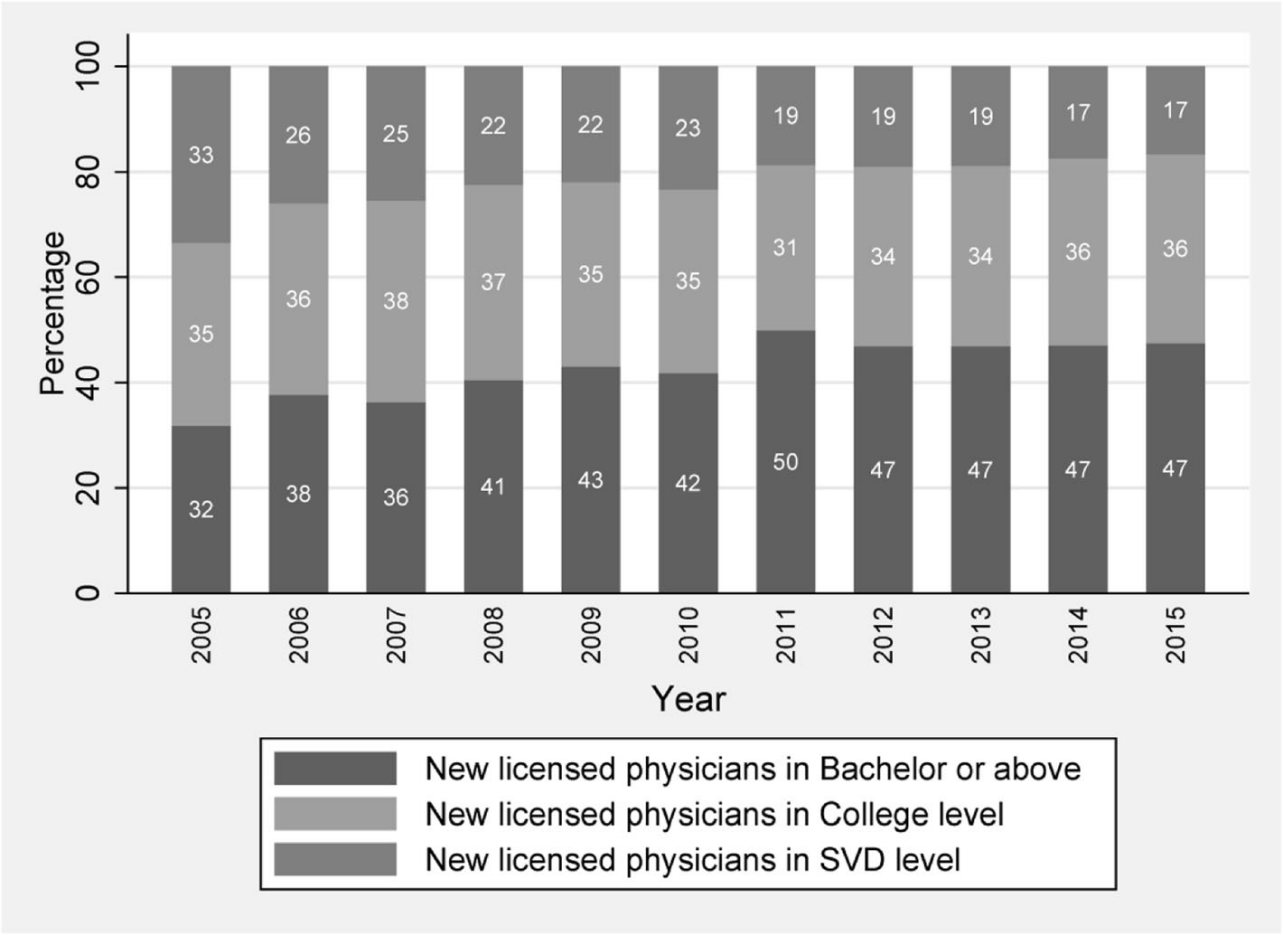
Educational distribution of newly licensed physicians, 2005–2015. Source: National Center for Medical Examination in China (various years)

The major progress of the current reform is to eliminate the medical programs in secondary-level education, so the major impact of the current reform is the shrinkage of stock of physicians with only secondary-vocational medical training. This effect was reflected by the change of educational distribution of newly licensed physicians during the past decade [12]. In specific, according to the data obtained from The National Center for Medical Examination in China, we investigate the changing pattern of the distribution of newly licensed physicians by education level between 2005 and 2015 (Fig. 3). For all data obtained from The National Center for Medical Examination, we excluded physicians whose education background cannot be identified, which only account for a very small part of the annual total number (< 0.1%). During this decade, the share of new physicians with middle education level (3-year college) remained almost constant in the range of 35 to 36%. However, the share of new physicians with only high school degree decreased substantially, from 33% in 2005 to 17% in 2015. By contrast, the share of newly licensed physicians with a bachelor degree or above increased from 32% in 2005 to 47% in 2015.

## Methods

Although China has initiated several rounds of reform in its medical education system, we need to explore whether doctors with different educational trainings will still coexist for a long time if the heterogeneity of medical education system in China has almost remained unchanged. We then analyze the effect of heterogeneity in medical education on the equity of health care. We further explore the impact of heterogeneity in medical education on the efficiency of health care market.

### Projection on growth of the medical practitioners

Based on the statistical description and the same data reported in both Figs. 2 and 3, we further predict the supply trend of newly licensed physicians during 2015–2035. We made four key assumptions in calculating the annual number of new supplied physicians in the following two decades. First, a total attrition rate in the medical practitioners is 3% a year for each year of the next 20 years. Second, we assume a consistent age structure among three educational groups. Third, it is reasonable to assume that total annual supplied number remains the same, because the government also aims to improve the quality of medical education and control for the quantity in the meantime according to the current policy [9, 13]. Fourth, the number of newly licensed doctors equal to the number of new hiring.

We manipulated our analysis by two scenarios. In scenario 1, we assume that supply structure stays the same as in 2015. In scenario 2, we assume that the reform package keeps the current path to eliminate 3-year SVD program; specifically, the ratio of new physicians in SVD level decreases by 1% for every year, while the ratio of new physicians in bachelor or above level increases by 1% for each year.

### The impact on equity

In our analysis, we define equity in terms of equal access to “good” doctors, which in turn is measured by whether they received good education training. The question we would like to explore is whether individuals living in different areas have equal access to good doctors. We first use the aggregate statistic at national level to show the distribution of physicians by regions and by education levels. We then use the individual data to show the effect of education level on the physician’s practicing location between the urban and rural sector. Our data was collected from a provincial survey of human resources for healthcare conducted between August and October 2009 that is a part of an overall investigation of the health system for the purpose of health sector reform in Fujian province (please refer to the cited paper for socio-demographic characteristics of the individual data). All human resources working for health organizations in Fujian province until 31 December 2008 were included as participants; therefore, this is almost a population-based dataset rather a sample selection [14].

In specific, we estimated a logistic regression model in the first column, and then, we estimated a fixed effects model in the second column. In the models, the dependent variable refers to a binary variable indicating the urban (= 1) and rural (= 0) settings for the hospitals, while independent variable “higher education” indicates three levels of education that is consistent through our study: secondary vocational diploma or below (= 1), college diploma (= 2), and bachelor or above (= 3). Rather than attempt to control for a multitude of county-level factors, we utilize county-level fixed effects. This absorbs factors that are time-invariant but that are likely to affect practice location, for example, physicians in counties with higher GDP are more likely to practice in rural area, or have high level of education.

### The impact on efficiency

First, we define the concept of efficiency. We analyzed the whole market included both demand side (patients) and supply side (physicians) by incorporating their interactions and dynamics. We refer the efficiency as the allocative efficiency which implies the optimal mix of outputs that achieves the goal of maximizing social welfare or health gain, which in turn is described by the right (optimal) point on production possibility frontier. In other words, allocative inefficiency is reduced if we alter the mix of services that the health system produces so that more health gains or higher social welfare can be achieved from a given resource endowment. For more information on economic efficiency, two cited papers can provide more perspectives [15, 16].

In this part, we will also utilize both theoretical reasoning and empirical analysis, to document evidence on the potential impact of heterogeneity in medical education on the efficiency of health care sector from three perspectives: (1) patients, (2) physicians, and (3) the system. In the empirical study, we still use the dataset of human resources for healthcare collected from Fujian province in 2009 to analyze the relationship between education level and the distribution of physician practice location among hospitals with various sizes. The hospital size in China has four clearly ranked categories, so we used an ordered probit analysis.

## Results

### Projection on growth of the medical practitioners

**Fig. 4:**
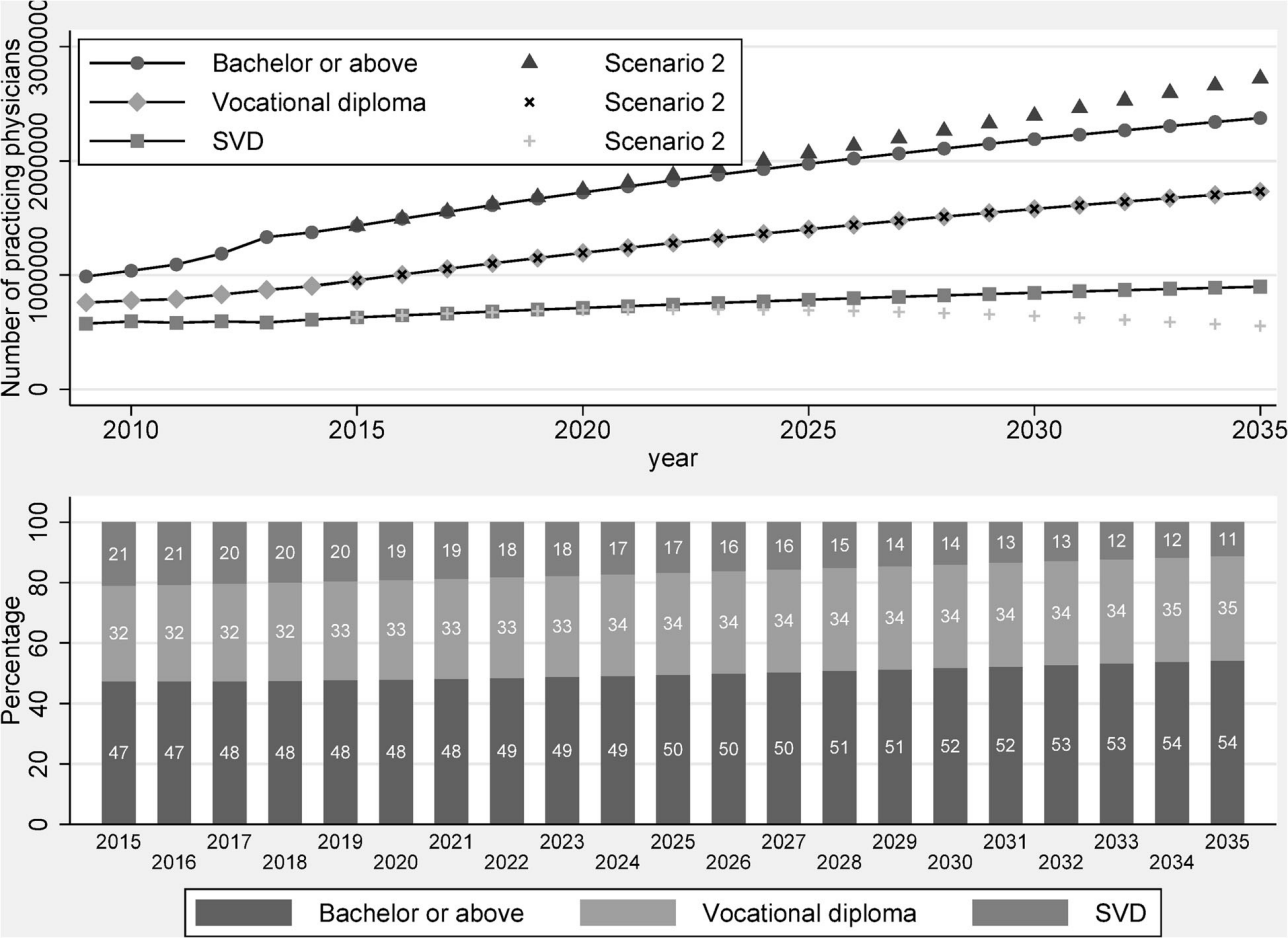
Predicted stock change of physicians by education level, 2010–2035. Notes: (1) The change of education structure is predicted based on scenario 2

Figure 4 reports the result of our prediction on the distribution of practicing physicians by education level between 2015 and 2035. The result shows that the share of physicians with a bachelor degree or above will increase over time, from 47% in 2015 to 54% in 2035. Although the proportion of physicians with only SVD training decreases over time, from 21% in 2015 to 11% in 2035, the physicians with vocational diploma still account for about one third of total number of practicing physicians in China. Putting together, even in 2035, physicians without bachelor degree still account for 46% of total number of practicing physicians, which indicates that the heterogeneity in China’s medical education is a long-run phenomenon and will not phase out over time. Thus, it is important to further investigate the impact of the heterogeneity in medical education on the equity and efficiency of health care market in China.

### The impact on equity

Given the evidence that there are three types of physicians (in terms of education training) in China, it is important to investigate whether the distribution of physicians across regions in China is correlated to the variations in physician’s education. There is an important implication for the equity in the delivery of health care service if physicians with better education training are more likely to concentrate in certain areas, such as urban areas or high-income cities.

As shown in Table 2, in 2005, near half of physicians who work in urban hospitals have a bachelor degree or above. This ratio increased to near two thirds in 2014. By contrast, in 2005, only about 4% of physicians who practices in rural township hospitals own a bachelor degree or above. This ratio increased to 11.9% in 2014. Putting together, this result suggests that physicians in the urban sector are more likely to receive high-quality training in their medical education while physicians in rural sector are more likely to receive a low-quality medical training.

**Table 2:** Distribution of doctors (including assistant doctors) by education level in 2005 and 2014

Educational level	Urban hospitals (%)		Rural township hospitals (%)	
	2005	2014	2005	2014
Bachelor or above	47.2	65.7	3.9	11.9
Vocational diploma	32.2	24.1	28.8	42.5
Secondary vocational diploma or below	20.6	10.2	67.3	45.6

Sources: Health Statistical Yearbook of China 2006 and 2015

The results from Table 3 show that all coefficients for age and gender are statistically insignificant at 1% level in the model (2). Compared to vocational diploma, respondents who had bachelor degree were found to be 19 (OR = 19.591, 95% CI = 17.809–21.552) times more likely to be associated with practicing in urban area. While compared to vocational diploma, respondents who had college diploma were found to be nearly three (OR = 2.699, 95% CI = 2.516–2.895) times more likely to be associated with practicing in urban area. Using the survey data obtained from Fujian province, the multivariate analysis indicates that physicians who have educational achievement higher than a bachelor degree are more likely to choose urban areas as the location of their practices, after controlling for all other variables.

**Table 3:** Regression analysis of the physician’s choice on the practice location between urban and rural areas

Dependent variable	Logit model: urban (= 1) vs. rural (= 0)	
	(1) Odds ratio	(2) Odds ratio
Higher education		
College diploma (dummy)	2.748*** [2.581, 2.926]	2.699*** [2.516, 2.895]
Bachelor degree or above (dummy)	34.480*** [31.665, 37.546]	19.591*** [17.809, 21.552]
Age	1.043*** [1.040, 1.047]	1.040*** [1.037, 1.043]
Male	0.435*** [0.408, 0.464]	0.474*** [0.441, 0.509]
Fixed effects		County
Number of observations	36 674	36 674
Log-likelihood function	− 1.59e+04	− 1.34e+04
Chi squared	10 950.468***	3 824.568***
Pseudo *R*-squared	0.257	0.356
BIC	31 759.125	26 834.060

Source: 2009 Fujian province database is a cross-sectional database that collected basic characteristics of human resources for health in all of the health institutes

Exponentiated coefficients are presented in the table; confidence intervals in parentheses

*** denote statistical significance at the 1% levels

**Fig. 5:**
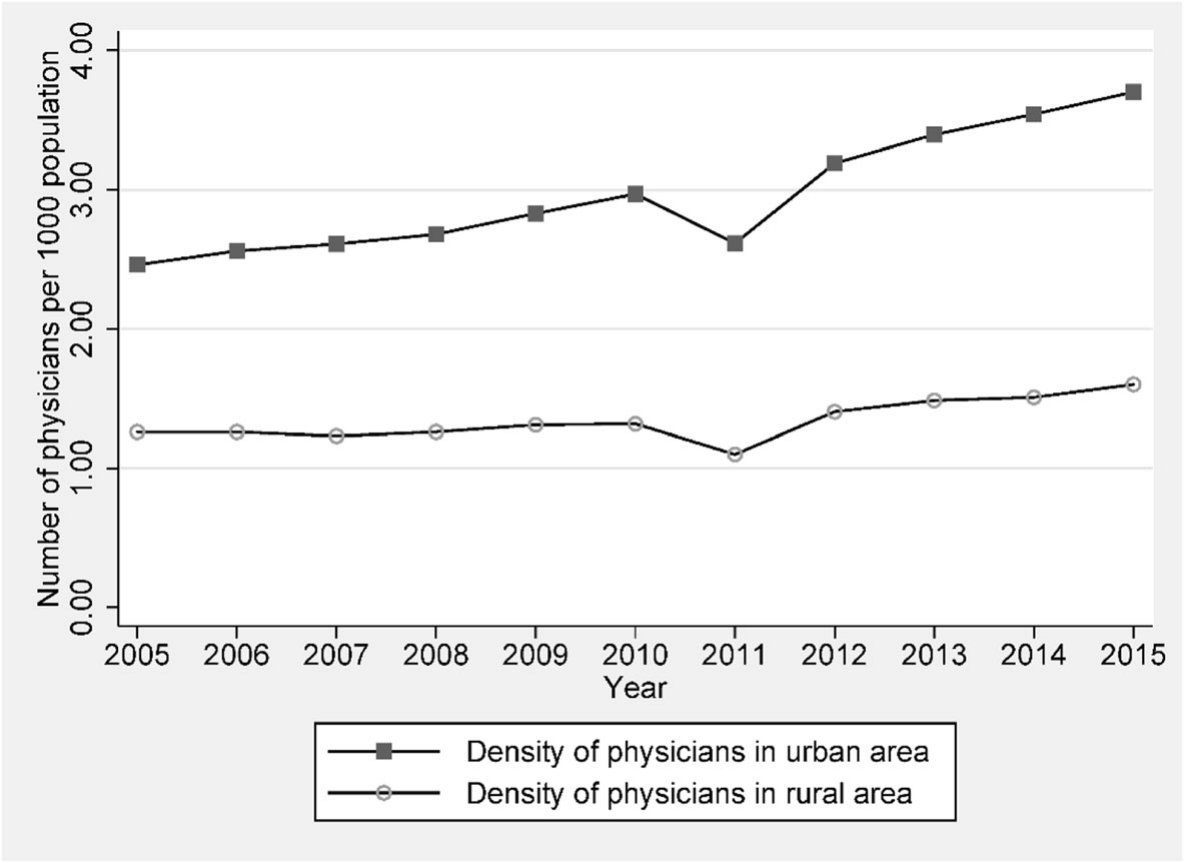
Time trend of physicians’ density between urban and rural area, 2005–2015. Source: Health Statistical Yearbook of China (various years)

Taken together, our results indicate that the heterogeneity in physician education training deteriorates the degree of geographical maldistribution of physicians across regions in China. As observed in other countries, rural areas tend to have lower physician density (number of physicians per 1 000 population) than urban areas. Figure 5 provides supporting evidence that the time trend of physician density in both urban and rural areas in China has been increasing from 2005 to 2015, while the number for the rural area has never been greater than 2, compared to that for the urban area ranging from 2.5 to 3.8. However, in China, the situation is getting worse in that there is a double disparity between rural and urban areas in the geographic maldistribution of physicians. Rural areas not only possess a disadvantage in terms of quantity (i.e., low physician density), but also have a disadvantage in terms of quality. That is, most well-educated physicians (with a bachelor degree or above) are concentrated in urban areas. Only a few well-educated physicians choose their practices in rural areas. As a result, the heterogeneity in medical education adds the fuels into the fire to amplify China’s regional inequality in health care. That is, rural and urban residents do not have an equal access to well-educated physicians.

### The impact on efficiency

In Table 4, the regressions use the hospital accreditation level as a proxy of hospital size, as in China hospital accreditation level is precisely based on the hospital size. Dependent variable refers to an indicator variable for small-level (= 1), fair-level (= 2), big-level (= 3), and large-level (= 4) hospitals. For all of the health institutions in China, they are categorized into four levels based on the organization size or the number of beds: township-level (number of beds, 1–20), county-level (number of beds 20–99), city-level (number of beds 100–499), and provincial-level (number of beds > 500) [17]. The independent variable we are concerned is still the “higher education”. The model (2) in the second column is still a model with county-level fixed effects.

**Table 4:** Regression analysis of the physician’s choice on the practice location between large and small hospitals

Dependent variable		Ordered probit model: hospital size	
		(1)	(2)
Higher education			
College diploma (dummy)		0.513*** (0.017)	0.559*** (0.022)
Bachelor degree or above (dummy)		1.759*** (0.016)	1.717*** (0.027)
Age		0.018*** (0.001)	0.019*** (0.001)
Male		− 0.381*** (0.013)	− 0.406*** (0.022)
Cut1	Constant	0.590*** (0.029)	− 24.252 (596.049)
Cut2	Constant	1.860*** (0.030)	− 19.456 (596.048)
Cut3	Constant	3.012*** (0.032)	− 5.556 (576.072)
Fixed effects			COUNTY
Number of observations		36 674	36 674
Log-likelihood function		− 4.03e+04	− 1.25e+04
Chi squared		14 936.433***	70 457.985***
Pseudo *R*-squared		0.156	0.738
BIC		80 659.477	26 115.339

Source: 2009 Fujian province database is a cross-sectional database that collected basic characteristics of human resources for health in all of the health institutes

*** denote statistical significance at the 1% levels. standard errors are reported in the parentheses

cut1—this is the estimated cutpoint on the latent variable used to differentiate *township-level hospital* from county-level, city-level, and provincial-level hospitals when values of the predictor variables are evaluated at zero

cut2—this is the estimated cutpoint on the latent variable used to differentiate *township-level* and *county-level hospital* from city-level and provincial-level hospitals when values of the predictor variables are evaluated at zero

cut3—this is the estimated cutpoint on the latent variable used to differentiate *township-level, county-level*, and *city-level hospitals* from provincial level hospitals when values of the predictor variables are evaluated at zero

As shown in Table 4, the intercept parameters are significantly different from each other so the four categories should not be combined into one. As observed in the education gradient between rural and urban areas, there is also an obvious education gradient in the distribution of physicians across health institutions. The results show that physicians who have education achievements higher than bachelor degree are more likely to practice in larger hospitals, after controlling for other variables (Table 4).

## Discussion

It will be a little bit ambitious to compare this study with the Flexner Report, an epoch-making study of medical education in the United States of America, written by Abraham Flexner and published in 1910 [18]. Our study, however, illustrated a similar story that was depicted and criticized by the Flexner Report a century ago. The fundamental message between two studies is that too many medical schools established and too many “doctors” trained, with significantly different admission and graduation standards. Our contribution is that we first pointed out the three-tier medical education program in China is a standard policy trade-off between quantity and quality in training health care professionals.

The advantage of such an education system is to increase the quantity of physicians supplied but at the expense of quality in education training. The previous researches pay more attention to the benefits of adopting a multi-tiered medical education system to the improvement of population health in the pre-reform era, such as the public health contribution of barefoot doctors to the decrease of mortality rate in China [19]. By contrast, little attention has been rigorously paid to the costs that the society pays by adopting a multi-tiered medical education system, especial for the post-reform era that China has experienced an epidemiologic transition from communicable to non-communicable diseases (NCDs). Our paper fills this gap by analyzing the impact of heterogeneity in medical education on both efficiency and equity in the delivery of health care system in China. Our analysis yields three important findings.

First, the geographic distribution of physicians in China is not uniform across physicians with different education training. We find the evidence that high-educated doctors are more likely to practice in urban areas while low-educated doctors are more likely to practice in rural areas. A number of evidence in both China and international show physicians’ human capital is most strongly associated with bad health outcomes through the mechanism of poor diagnosis decision [20–22]. Given that high-educated doctors are in a better position to provide high-quality health care, the concentration of high-educated physicians in urban areas indicates that there is an inequality in the delivery of health care services between rural and urban areas in that rural residents are in a disadvantage position to receive the high-quality health care services.

Second, we also find the evidence that high-educated doctors are more likely to be hired by larger hospitals, which in turn add the fuel to foster the hospital-center health care system in China as patients choose large hospitals to chase good doctors. However, chasing good doctors in large hospitals has a price to pay: deteriorating the trust relationship between patients and their doctors. This is because the large patient volumes put patients in a very difficult position to build the long-term relationship with their doctors.

Third, the heterogeneity in medical education also creates both adverse selection and moral hazard to the health care market in China. On the one hand, the heterogeneous education system deters the entry of high-competent doctors into the health care market as the regulated market only pay the average salary to all doctors, and hence, good doctors do not receive a good pay. On the other hand, the heterogeneity in medical education deters the incentive for continuing education and new investment on human capitals among the existing doctors as the rate of return of such an investment is low, which in turn removes incentives to develop credentials or qualifications.

### Perspectives from economic theories

In this subsection, we further discuss the impact of heterogeneity in medical education on the efficiency of health care sector from three perspectives: (1) patients, (2) physicians, and (3) the system.

From the patients’ perspective, the heterogeneity in medical education imposes additional search and informational costs to seek for good doctors. China, like many other Asian countries including Japan and South Korea, has a closed-staff hospital system whereby physicians practicing in hospitals are employees of hospitals [23]. In addition, few physicians in China practice in their own clinics. Given the fact that public hospitals and other public health institutions account for about 90% of market share in China’s health sector, physicians serving as state employees are just similar to civil servants who in turn are subject to several government regulations, such as headcount quota and the link of physician licensing with employed institutions. As a result, physicians are restricted to have the mobility opportunity across health institutions.

Due to the asymmetric information in health care market, patients often do not possess full information to know who good doctors are. Rather, according to our results reported at Tables 3 and 4, patients only have limited information to know that good doctors (as measured by sufficient education training) are in large hospitals and in urban sectors. Thus, patients need to incur two additional costs to find a good doctor: search cost and time cost. Search cost is also called the cost of frictional market that has been extensively studied by the search and matching theory [24, 25]. In the theory, any patient exercising rational efforts keeps on searching for better physicians till the moment that extra cost of searching is less than the marginal benefits which the physician provides. Search cost consists of external and internal costs [26]. External costs refer to the monetary costs of searching the quality information provided by physicians and the opportunity cost of searching time. Internal costs refer to mental efforts of quality information searching, sorting, and integration. Undoubtedly, a market with heterogeneous physicians will significantly increase the search cost, as no minimum quality assurance guarantee exists in the market.

Since patients need to spend more time to chase good doctors, which in turn imposes a higher “time costs” to the patients and their family. This incentive in turn creates a hospital-centered health system in China as good doctors are locked in larger hospitals located in urban areas. As a result, the associated time costs spent on searching for and get access to good doctors in larger hospitals, such as additional time costs allocated to travel and long waiting time, are efficiency loss in China’s health system.

In addition, the hospital-centered health system put patients in a difficult position to build the trust and long-term relationship with their doctors. This is because higher search cost will definitely yield higher transaction costs, in which one of the most serious results is patients’ low trust in physicians. Low trust is sometimes considered as the biggest threat to physician-patient relationships. As the time cost is very high to get access to doctors in larger hospitals, especially for patients coming from the rural areas, the patient-physician relationship is just like “one-time buyers” instead of “repeat customers.” Furthermore, given the high patient volumes in larger hospitals, physicians only can spend a very short time with each patient. Taken together, chasing a good doctor in larger hospitals end up with the attenuation of the trust relationship between patients and physicians, as observed by the increasing number of medical malpractice litigations in recent years [27]. To mitigate the threat of medical malpractice litigation, physicians in turn adopt the strategy of defensive medicines, such as over prescription [28], which also end up with rising health care cost and imposing the efficiency loss to the health care system.

From the perspective of physicians, the heterogeneity of medical education imposes two types of efficiency loss to health care market: adverse selection and moral hazard. According to the model developed by Akerlof in 1970s, high-quality doctors will not enter the market if patients do not observe physician’s ability that only physician knows and physicians’ fees will not depend on the quality of services [15]. There is an efficiency loss in terms of the reduction in social welfare when the quacks prevail in the market and the average quality of health care decreases. It is reasonable that the case is especially serious for a market in full of physicians with heterogeneous education training.

Shapiro assumes service quality derives directly from endogenous human capital investment on the one hand and affects physicians’ reputations on the other hand [16]. There is a quality deterioration effect if asymmetric information on the investment between physician and patient exists in the market. Specifically, the quality services will be primarily undersupplied because of the imperfect reputation mechanism or moral hazard problems among heterogeneous physicians.

In addition, health care market is generally a twofold market with an upstream market, the physicians market, and a downstream market, the market for delivering health care services. Therefore, the cost of upstream market will be transmitted to the downstream market [29]. In this case, we assume low-quality doctors have a smaller market share or allocation size than high-quality doctors, e.g., smaller number of inpatient workloads among low-quality doctors. Then, the group of quack doctors due to physicians’ heterogeneity in training which in turn have strong motivations to provide a large amount of unnecessary health care through physician induced demand instead of appropriate diagnoses and treatments. Essentially, it is still the cost due to asymmetric information as mentioned in the third stream of costs; however, it is slightly different from the above as this kind of cost mainly focuses on the health service provision.

From the perspective of health system as a whole, the heterogeneity in medical education distorted the specialty mix in health care market, which in turn leads to a higher cost of health service delivery. A series of studies have shown that competent primary care providers can enhance the performance of the health care system as a whole [30, 31]. The heterogeneity of physicians substantially worsens the specialty mix because physicians with higher education are more likely to be specialists in hospitals. As a result, the shrinkage of doctors at the primary care setting combined with low human capital deteriorates the human resources for primary health. In addition, when the “low-quality” GP cannot act their role in the division of labor, the search costs further increase due to a lack of information on “gate-keeper” of health care system.

### The options of mitigation policy

Taken together, the heterogeneity of medical education in China has a strong negative impact on the performance of health sector in terms of reducing equity and efficiency. Since it will take a long time for China to achieve the uniform quality standard in medical education, it is important to seek for other policy options to mitigate the current negative impacts arising from the heterogeneous education system for medical doctors. Based on the abovementioned theoretical reason and empirical evidence, we discuss several policy options as follows.

It is obvious that the current “The Law for Licensing Medical Practitioner” issued in 1999 needs to be modified. The law allows medical graduates with non-bachelor degree to become assistant doctors, and then, they have the chance to obtain the full doctor licensure after they accumulate certain years of work experience and pass an examination. Our study recommended to policymakers to adopt the globally agreed-upon definition of the doctor/physician and to restrict its occupational regulation. The latest version of the International Labor Organization’s International Standard Classification of Occupations (ISCO) is published in 2012 (ISCO-08) (please see section #221: Medical doctor in page 125, http://www.ilo.org/wcmsp5/groups/public/%2D%2D-dgreports/%2D%2D-dcomm/%2D%2D-publ/documents/publication/wcms_172572.pdf).

As there is significant disparity in doctors’ education training between rural and urban areas as well as between large and small hospitals, it is beneficial to establish a learning health system that doctors with low education attainment can learn from doctors with high education attainment. This is technically feasible by adopting the health information and communication technology to integrate doctors in different locations and different institutions into a learning network. This learning network provides a platform to close the knowledge gap between rural and urban areas as well as between large and small hospitals. Specifically, physicians in rural and/or small hospitals can learn from physicians in urban and/or large hospitals through the development of clinical pathway, evidence-based care, and tele-medicine.

In addition, it has been widely recognized that reforming payment system is the key to increase the efficiency of the health care system [32]. Current payment system in China is a volume-based, which rewards physicians according to the volume of health care services they provided, such as number of outpatient visits, number of inpatient admissions, and number of prescription drugs, instead of the outcomes they achieved. During the past decades, many countries have adopted a series of reform package to transform their payment system from the traditional volume-based to value-based [33]. The value-based payment system emphasizes the value, which is defined as health outcomes for the money spent, and is in a better position to reward the good doctors than the traditional volume-based payment system as the value-based payment system provides powerful incentive to encourage physician to value better outcome instead of valuing quantity of services [34]. Under the traditional payment system, many quality aspects of health care services, such as time, diligence, care, and attentiveness, are not reimbursed by the payer [32]. By contrast, physicians’ additional efforts and time inputs as well as additional education training, which are strongly related to outcome, are reimbursed by the payer under the value-based payment system. Thus, good doctors who emphasize patient outcomes will receive good pays. Given that reforming the medical education system takes a longer time (may be a generation) than reforming payment system (e.g., a few years), a viable policy option for China in the short run is to mitigate the efficiency loss in health care market through the payment system reform.

## Conclusion

Like the Flexner Report over a century ago, this study pointed out one thing in common between two countries across the history of 100 years, which is featured by too many medical schools established and too many “doctors” trained, with significantly different admission and graduation standards. This paper reviews the historical background of multi-tiered medical education in China and investigates its impact on the performance of health care market. Although the multi-tiered medical education system enjoyed the benefits of training doctors in a quick way, it also pays the price to lower the overall quality of physician training and further the quality of health care. Previous literature has paid more attention on the contribution of low-quality physicians in achieving the remarkable public health gain, such as the legend of barefoot doctors in rural China. By contrast, there is no virtual study to pay attention on the cost of such multi-tiered education system to the modern society in China.

The contribution of our paper is to fill the literature gap by providing the quantitative evidence on the cost side of the multi-tiered medical education system. Based on both national aggregate statistics and provincial survey data, we find evidence that high-quality physicians (in terms of higher education attainment) are more likely to concentrate in urban areas and in larger hospitals, which in turn create significant negative impacts on the performance of health care sector in China. On the one hand, the unequal distribution of high-quality physicians between urban and rural areas amplifies the regional inequality in the access to high-quality care in China. On the other hand, the unequal distribution of high-quality physicians across health institution also contributes to the formation of hospital-centered health care system, which in turn imposes various types of efficiency loss to the health care market in China, including a higher time costs of accessing health care services and lower trust relationship between physicians and their patients.

Our analysis also indicates that the multi-tiered medical education system will not phase out in the near future. As a result, it is important to find effective policy options to mitigate the social costs imposed by the multi-tiered medical education system. Our paper highlights two important policy tools in addressing the equity and efficiency issues arising from multi-tiered medical education. The first option is to use information and communication technologies to integrate high- and low-quality physicians into a learning network. The second option is the payment system reform to shift the mechanism of rewarding physicians from volume-based to value-based. Both policy tools have sound theoretical rationales and empirical evidence, but more studies are needed for the implementation of these two policies. There are also recommendations for future studies; it is important to extend our perspectives on the impacts of heterogeneity in medical education on the health care market to other countries, especially those developing countries where the governments hope to modernize the health care markets.

## Data Availability

The data is not available for this project.
